# Caesarean section without medical indications is associated with an increased risk of adverse short-term maternal outcomes: the 2004-2008 WHO Global Survey on Maternal and Perinatal Health

**DOI:** 10.1186/1741-7015-8-71

**Published:** 2010-11-10

**Authors:** JP Souza, AM Gülmezoglu, P Lumbiganon, M Laopaiboon, G Carroli, B Fawole, P Ruyan

**Affiliations:** 1 UNDP/UNFPA/WHO/World Bank Special Programme of Research, Development and Research Training in Human Reproduction, WHO, Geneva, Switzerland; 2 Department of Obstetrics and Gynaecology, Faculty of Medicine, Khon Kaen University, Khon Kaen, Thailand; 3 Department of Biostatistics and Demography, Faculty of Public Health, Khon Kaen University, Khon Kaen, Thailand; 4 Centro Rosarino de Estudios Perinatales Rosario, Argentina; 5 Department of Obstetrics and Gynecology, University College Hospital, Ibadan, Nigeria; 6 Maternal and Child Health Department, Public Health School, Peking University, Beijing, China

## Abstract

**Background:**

There is worldwide debate about the appropriateness of caesarean sections performed without medical indications. In this analysis, we aim to further investigate the relationship between caesarean section without medical indication and severe maternal outcomes.

**Methods:**

This is a multicountry, facility-based survey that used a stratified multistage cluster sampling design to obtain a sample of countries and health institutions worldwide. A total of 24 countries and 373 health facilities participated in this study. Data collection took place during 2004 and 2005 in Africa and the Americas and during 2007 and 2008 in Asia. All women giving birth at the facility during the study period were included and had their medical records reviewed before discharge from the hospital. Univariate and multilevel analysis were performed to study the association between each group's mode of delivery and the severe maternal and perinatal outcome.

**Results:**

A total of 286,565 deliveries were analysed. The overall caesarean section rate was 25.7% and a total of 1.0 percent of all deliveries were caesarean sections without medical indications, either due to maternal request or in the absence of other recorded indications. Compared to spontaneous vaginal delivery, all other modes of delivery presented an association with the increased risk of death, admission to ICU, blood transfusion and hysterectomy, including antepartum caesarean section without medical indications (Adjusted Odds Ratio (Adj OR), 5.93, 95% Confidence Interval (95% CI), 3.88 to 9.05) and intrapartum caesarean section without medical indications (Adj OR, 14.29, 95% CI, 10.91 to 18.72). In addition, this association is stronger in Africa, compared to Asia and Latin America.

**Conclusions:**

Caesarean sections were associated with an intrinsic risk of increased severe maternal outcomes. We conclude that caesarean sections should be performed when a clear benefit is anticipated, a benefit that might compensate for the higher costs and additional risks associated with this operation.

## Background

Caesarean sections performed appropriately and following an appropriate medical indication are potentially life-saving procedures. In this context, the provision of timely and safe caesarean sections in high maternal mortality countries is a major challenge faced by local health systems [1]. At the same time, in many settings, women are increasingly undergoing caesarean sections without any medical indication which may contribute to the worldwide secular trend towards higher rates of caesarean sections [2,3]. Over the last two decades, there has been a debate about the appropriateness of caesarean sections performed due to maternal request or following the indication of health care professionals but without a clear medical reason for this surgical procedure. Safety, costs, women's rights and wishes, maternal and professional satisfaction have been elements of this debate [4-10].

One factor that certainly favoured the liberalization of caesarean section in clinical practice has been the perception of caesarean section as a generally safe procedure, despite the increased costs associated with it. However, the assessment of the intrinsic risk of caesarean sections is complicated by substantial limitations in the existing medical literature [11,12]. Strong evidence would be provided by a well-designed randomized controlled trial, in which healthy women without co-existing medical conditions would be allocated to either intention to deliver by elective caesarean section or expectant management [13]. Obviously, ethical constraints prevent such a trial. In other designs, the scientific community has been struggling with indirectness, imprecision due to sample size limitations and paucity of data, or difficulties in disentangling confounders and effect modifiers in order to establish clear associations between caesarean sections and the occurrence of severe maternal outcomes.

In this context, the World Health Organization Global Survey on Maternal and Perinatal Health (WHOGS) provides evidence on the relationship between mode of delivery and maternal and perinatal outcomes. The WHOGS is a large cross-sectional study conducted in 24 countries around the world between 2004 and 2008. The project has been implemented in three continental steps, and each continental analysis has shown an association between caesarean section and an increased risk of adverse maternal outcomes [5-7,14,15]. In the present three continental analyses we aimed to further investigate the relationship between caesarean section and severe maternal outcomes; more specifically, we wanted to assess the intrinsic risk of caesarean sections with a special focus on those without medical indication.

## Methods

### Study design

Methodological details of the global survey have been published elsewhere [14]. Briefly, this is a multicountry, facility-based survey that collected data for all delivering women in randomly selected facilities from randomly selected countries. A stratified multistage cluster sampling design was used to obtain a sample from countries and health institutions worldwide. Countries in the WHO regions were further grouped according to adult and under-five infant mortality. From each of these sub regions, four countries were selected, with probability proportional to population size. A total of 54 countries were selected, but owing to financial and practical constraints, the study was implemented in the selected countries of three continents: Africa, the Americas and Asia. For the same reasons, developed countries were excluded (unless they volunteered to participate with their own funds, for example, Japan). Few other countries (for example, the Democratic People's Republic of Korea, Ethiopia, Haiti, Indonesia) could not participate due to country refusal, security issues or other reasons. A total of 24 countries took part in the study. In each country, two regions or provinces, in addition to the capital city, were randomly selected with probability of selection proportional to their size. Once a province had been selected, we obtained a census of all facilities within the province with more than 1,000 births per year and those reported as able to perform caesarean sections. If there were more than seven facilities, seven were randomly selected with probability of selection proportional to the number of births per year. If there were fewer than seven facilities, all were selected. In each of the selected institutions, we studied all women admitted for delivery during three months in institutions with 6,000 or fewer expected deliveries per year and during two months in those with more than 6,000 expected deliveries per year.

Data collection took place during 2004 and 2005 in Africa and the Americas and during 2007 and 2008 in Asia. We obtained written permission from all ministries of health of the participating countries and the directors of the selected facilities. We obtained data for all individuals from medical records and participants were not identified. The Ethics Review Committee of WHO and of each country independently approved the protocol.

### Data collection

Data were collected for institutions and for individuals. For institutions, the hospital coordinator completed a form in consultation with the director or head of obstetrics. Data included characteristics of maternal and perinatal care, including the availability of laboratory tests; anesthesiology resources; services for intrapartum care, delivery, and care of the newborn baby; and presence or absence of basic emergency medical and obstetric care facilities, intensive care units (ICUs), and human and training resources. For individuals, data were obtained from women's medical records to complete a two-page form. Individual data included demographic characteristics, maternal risk, current pregnancy, method of delivery, and outcomes (maternal and perinatal) up to hospital discharge. All women giving birth at the facility during the study period were included. Trained staff reviewed medical records of all women and their babies before discharge from the hospital, and abstracted data daily to their forms for individual data collection. The hospital coordinator supervised data collection, resolving or clarifying unclear medical notes before forms were sent for data entry. Attending staff updated incomplete records before discharge. Criteria for data abstraction were defined in the manuals of operations, which were available for staff training and monitoring of data quality, keeping to a minimum the need for judgment and interpretation. The manual contained definitions of all terms used and synonyms of medical and obstetric terms, and described questions and corresponding answers. Shortly after collection, data were entered at the country, provincial or facility level in a web-based system (MedSciNet AB, Stockholm, Sweden).

### Statistical analysis

In this three-continent analysis, the continental databases were merged into a global database. Then, frequencies were used to describe modes of delivery for each country and facility characteristics as well as characteristics of mothers and babies for each group's mode of delivery (including caesarean section without medical indication). The main outcome was severe maternal outcome, defined as the occurrence of any of the following conditions: death, admission to ICU, blood transfusion or hysterectomy within the seven days following birth. The severe perinatal outcomes considered were fetal death, neonatal mortality up to hospital discharge limited to the first week of life, and stay of more than or equal to seven days in the neonatal intensive care unit. Univariate analysis followed by generalized linear and latent mixed models (GLLAMM) for multilevel analysis was performed to study the association between each group's mode of delivery and the severe maternal and perinatal outcome using the GLIMMIX procedure in SAS (version 9.1 SAS Institute Inc., SAS Campus Drive, Cary, North Carolina, USA). This procedure was intended to account for clustering effects within facilities and the analysis was adjusted for possible confounding factors and effect modifiers, including individual and institutional characteristics. Among the institutional characteristics, a hospital complexity index was used to assess the hospital capacity in terms of providing essential health services, emergency obstetric care and human resources. This hospital complexity index was used in previous Global Survey analysis and was detailed elsewhere [14]. Risks of maternal and perinatal outcomes associated with modes of delivery (including caesarean section without medical indications) were presented by adjusted Odd Ratios (Adj OR) with corresponding 95% Confidence Intervals (95% CI). Heterogeneity between countries was explored through simple regional stratification and adjusted country-based forest plots. Two subgroup analyses were conducted, one excluding all women with morbidities, complications, multiple pregnancies, breech presentations and previous caesarean sections and another of neonates with breech presentation.

## Results

**Figure 1:**
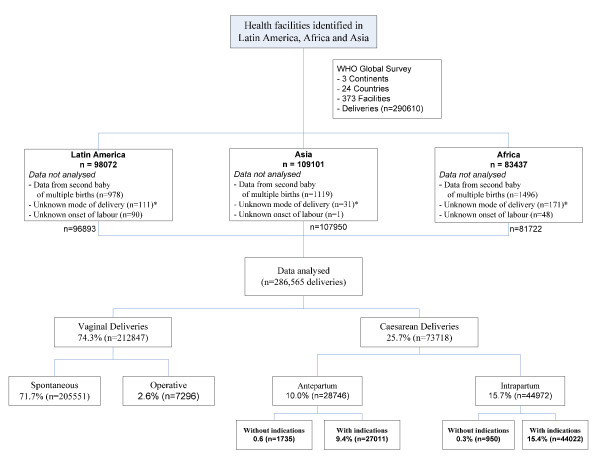
**Study profile**. *Includes laparotomy for ruptured uterus.

A total of 24 countries and 373 health facilities participated in the WHO Global Survey on Maternal and Perinatal Health, collecting information on 290,610 deliveries. The study was implemented in seven African countries (131 health facilities, 83,437 participants), eight Latin American countries (120 health facilities, 98,072 participants) and nine Asian countries (122 facilities, 109,101 participants). Most of the facilities were located in urban areas (n = 275) and of low complexity (n = 280). Data from the second or high order baby in multiple pregnancies and from participants with unknown mode of delivery or unknown onset of labour were not analysed, resulting in 286,565 participants included in the present analysis. Figure 1 presents the study profile, while Table 1 shows the total number of deliveries and facilities by country, and percentages of deliveries by mode and country. African countries had the highest rates of spontaneous vaginal delivery while Asian countries tended to have higher rates of operative vaginal delivery. The overall caesarean section rate was 25.7% and the Chinese health facilities had the highest caesarean section rates (average for Chinese health facilities: 46.2%). A total of 1.0 percent of all deliveries had caesarean sections without medical indications, either due to maternal request or in the absence of other recorded indications. In 23 out of 24 countries, the overall proportion of women delivering by caesarean section without medical indication ranged from 0.01 to 2.10%. In the Chinese institutions participating in this survey this figure was exceptionally higher at 11.6%. A total of 63% of all caesarean sections without medical indications were performed in Chinese health facilities (n = 1,689/2,685 caesarean sections without medical indications; 11.6% of all deliveries in China). Table 2 presents characteristics of women by mode of delivery. Women delivering by caesarean section antepartum without medical indications had some distinctive features: a higher proportion of married women, fewer adolescents, more educated and more primiparous women, and less frequent antecedent of low birth weight and fetal and neonatal death in a previous pregnancy. A proportion of women having caesarean sections without a medical indication had some other co-morbidities, which, nevertheless, were not considered as the indication for the caesarean section.

**Table 1:** Total number of deliveries and facilities by country, and the percentages of deliveries by mode and country

	Vaginal Delivery		Caesarean section without indications		Caesarean section with indications		Total deliveries	Total facilities
	Spontaneous	Operative	Antepartum	Intrapartum	Antepartum	Intrapartum		
	%	%	%	%	%	%	n	n
***Africa***								
**Algeria**	80.5	5.9	0.04	0.1	5.9	7.5	15,578	18
**Angola**	97.7	0.8	0.1	0.02	1.0	0.5	6,322	20
**D.R. Congo**	84.8	2.3	0.1	0.1	1.4	11.4	8,804	21
**Kenya**	82.9	1.5	0.1	0.5	3.2	11.8	19,968	20
**Niger**	91.5	3.2	0.02	0.2	0.4	4.8	8,275	11
**Nigeria**	81.1	4.4	0.1	0.3	3.6	10.5	8,939	21
**Uganda**	85.4	1.2	0.1	0.3	2.1	10.9	13,836	20
								
***Americas***								
								
**Argentina**	62.1	2.8	0.2	0.1	17.8	17.0	10,689	14
**Brazil**	68.5	2.0	0.3	0.2	15.4	13.6	15,129	19
**Cuba**	61.7	2.7	0.02	0.1	16.6	18.8	12,642	17
**Ecuador**	59.2	0.5	0.1	0.6	7.8	31.8	12,414	18
**Mexico**	60.4	1.9	0.1	0.1	15.8	21.8	20,883	21
**Nicaragua**	69.1	0.1	0.1	0.2	7.7	22.8	5,636	8
**Paraguay**	57.3	0.8	1.2	0.9	18.1	21.7	3,460	17
**Peru**	65.4	0.6	0.01	0.0	13.2	20.8	16,040	17
								
***Asia***								
								
**Cambodia**	77.6	7.7	0.04	0.2	2.6	11.8	5,565	5
**China**	52.6	1.2	9.3	2.3	19.7	14.9	14,541	21
**India**	79.4	2.9	0.1	0.2	3.6	13.8	24,682	20
**Japan**	74.1	6.1	0.1	0.0	13.9	5.9	3,300	10
**Nepal**	76.0	3.8	0.04	0.1	6.2	14.0	8,489	8
**Philippines**	78.4	2.8	0.04	0.1	8.0	10.3	13,295	6
**Sri Lanka**	65.9	3.5	0.5	0.2	20.2	9.8	15,024	14
**Thailand**	61.6	4.3	0.3	0.1	13.4	20.2	9,745	12
**Vietnam**	62.2	2.2	0.2	0.8	4.3	30.2	13,309	15
								
**Total**	71.7	2.6	0.6	0.3	9.4	15.4	286,565	373

**Table 2:** Selected maternal characteristics and mode of delivery (%)

	Vaginal delivery		Caesarean section without indications		Caesarean section with indications		Total
	Spontaneous	Operative	Antepartum	Intrapartum	Antepartum	Intrapartum	
	N = 205,551	N = 7,296	N = 1,735	N = 950	N = 27,011	N = 44,022	N = 286,565
Marital status (single)	13.5	9.3	2.7	6.0	11.8	10.7	12.7
Maternal age							
≤16 years	2.5	2	0.1	1.9	1.4	2.4	2.3
16 to 35	87.8	85.7	89.4	85.9	80.1	86	86.8
≥35	9.7	12.3	10.6	12.3	18.5	11.6	10.9
Years of education							
<7	30.9	28.4	11.4	20.5	18.4	24.7	28.6
7 to 12	57.1	54.9	59.0	56.1	59.4	57.6	57.4
>12	12	16.7	29.7	23.4	22.2	17.7	14.1
Primiparous	41.2	57.3	73.3	58.8	37.6	49.7	42.8
							
**Previous pregnancy**							
Low birth weight	10.0	14.1	3.1	5.4	14.9	10.1	10.5
Fetal or neonatal death	2.0	2.4	1.6	3.6	2.6	2.6	2.1
Caesarean delivery in the last pregnancy	2	5.9	6.7	11.9	39.3	23.5	9
							
**Current pregnancy**							
Induced labour	9.9	15.4	0.0	13.9	0.0	13.9	9.7
Prelabour rupture of membranes	9.4	13.2	8.4	16.0	8.7	15.3	10.3
Pregnancy induced hypertension	2.8	5.3	2.9	3.2	8.5	5.5	3.8
Pre-eclampsia	1.6	3.2	1.3	1.3	8.9	4.8	2.8
Eclampsia	0.2	0.8	0.1	0.3	1	0.6	0.4
Chronic hypertension	0.6	1	0.2	0.2	2.4	1.2	0.8
Vaginal bleeding in second half of pregnancy	0.9	1.2	0.6	0.9	3.6	2	1.3
Suspected fetal growth impairment	0.8	1	0.3	0.7	2.5	1	1
Breech or other non-cephalic presentation	1	25.1	0	0	14.7	12.6	4.7
Referred for complication related to pregnancy	18.8	23.9	9.1	14.2	34	28.8	21.8
HIV	0.8	0.5	0.5	1.4	1	0.9	0.8
Cardiac/Renal diseases	0.3	1	0.5	0.5	1.1	0.5	0.5
Chronic respiratory conditions	0.5	0.7	0.3	0.2	1	0.6	0.6
Diabetes mellitus	0.4	0.7	1.5	0.3	2.6	1	0.7
Malaria	3.3	2.7	0.2	1.3	0.4	1.1	2.6
Sickle cell anaemia	0.3	0.1	0.0	0.3	0.5	0.4	0.3
Severe anaemia	0.5	0.8	0.1	0.5	0.8	0.5	0.6
Pyelonephritis or urinary infection	5.9	4.4	0.6	5.4	8.4	8.6	6.5
Other medical conditions	5.3	5.3	10.2	5.7	12.6	8.2	6.5

Table 3 presents the risk of severe maternal outcomes by mode of delivery. Overall, the prevalence of severe maternal outcomes was 37 cases/1,000 deliveries. In addition, compared to spontaneous vaginal delivery, all other modes of delivery presented an association with increased risk of death, admission to ICU, blood transfusion and hysterectomy, including antepartum caesarean section without medical indications (Adjusted Odds Ratio (Adj OR), 5.93, 95% Confidence Interval (95% CI), 3.88 to 9.05) and intrapartum caesarean section without medical indications (Adj OR, 14.29, 95% CI, 10.91 to 18.72). Table 4 presents the stratification of the combined severe maternal outcomes by region, showing that in all regions there was a trend towards an increased risk associated with caesarean sections without indication. This association is stronger in Africa, compared to Asia and Latin America. The adjusted country level analysis and the resulting country-based forest plots were limited by the low frequency of severe maternal outcomes: in various countries the outcome data (adjusted OR) became zero or non estimable after adjustment for the factors listed at the footnote of Table 3. Overall, the results at the country-level were more imprecise but a trend towards an increased risk has been observed with no country presenting statistically significant results favoring caesarean section. In the subgroup of women without any recorded morbidity including multiple pregnancies, breech presentations and previous caesarean sections, caesarean sections without indications were compared to spontaneous vaginal births. A total of 1,412 women having caesarean sections were compared to 118,742 women having a spontaneous vaginal delivery: 26 women having caesarean sections and 879 women having vaginal deliveries presented severe maternal outcomes (OR = 2.52; 95% CI 1.70 to 3.73).

**Table 3:** The relationship between mode of delivery and severe maternal outcomes (that is, death, admission to ICU, blood transfusion, hysterectomy)

Maternal outcomes	n/N (%)	Adjusted OR [95%CI]
Death*		
Spontaneous (reference)	196/205,551 (0.10)	1
Operative	34/7,296 (0.47)	2.9 (1.84 to 4.56)
Antepartum without indications	**0/1,735(0)**	Not estimated
Intrapartum without indications	**2/950(0.21)**	3.21 (0.78 to 13.2)
Antepartum with indications	**36/27,011(0.13)**	1.51 (0.97 to 2.33)
Intrapartum with indications	**74/44,022(0.17)**	1.7 (1.24 to 2.33)
Admission to ICU**		
Spontaneous (reference)	1,189/205,551 (0.58)	1
Operative	172/7,296 (2.36)	2.27 (1.89 to 2.73)
Antepartum without indications	**22/1,735(1.27)**	30.75 (18.12 to 52.17)
Intrapartum without indications	**67/950(7.05)**	58.85 (41.46 to 83.52)
Antepartum with indications	**1,741/27,011(6.45)**	63.4 (56.32 to 71.36)
Intrapartum with indications	**3,457/44,022(7.85)**	51.3 (46.56 to 56.59)
**Blood transfusion**^**†**^		
Spontaneous (reference)	1,940/205,303 (0.94)	1
Operative	198/7,287 (2.72)	2.11 (1.79 to 2.48)
Antepartum without indications	**9/1,735(0.52)**	1.79 (0.91 to 3.52)
Intrapartum without indications	**18/950(1.89)**	3.7 (2.24 to 6.1)
Antepartum with indications	**892/26,967(3.31)**	3.75 (3.39 to 4.14)
Intrapartum with indications	**1,475/43,928(3.36)**	3.85 (3.55 to 4.16)
**Hysterectomy**^**††**^		
Spontaneous (reference)	82/205,299 (0.04)	1
Operative	11/7,278 (0.15)	3.38 (1.73 to 6.59)
Antepartum without indications	**0/1,733(0)**	Not estimated
Intrapartum without indications	**4/949(0.42)**	13.53 (4.79 to 38.2)
Antepartum with indications	**100/26,916(0.37)**	6.11 (4.38 to 8.52)
Intrapartum with indications	**118/43,889(0.27)**	6.68 (4.91 to -9.09)
**Severe Maternal Outcomes**^**†††**^		
Spontaneous (reference)	3,147/205,551 (1.53)	1
Operative	346/7,296 (4.74)	1.84 (1.62 to 2.1)
Antepartum without indications	**28/1,735(1.61)**	5.93 (3.88 to 9.05)
Intrapartum without indications	**86/950(9.05)**	14.29 (10.91 to 18.72)
Antepartum with indications	**2,452/27011(9.08)**	13.28 (12.3 to 14.34)
Intrapartum with indications	**4,523/44,022(10.27)**	12.05 (11.33 to 12.82)

*Total number of years attended school, birth weight, Pregnancy induced hypertension or preeclampsia or eclampsia or chronic hypertension, suspected fetal growth impairment, vaginal bleeding in second half of pregnancy, Breech or other non-cephalic presentation, referred for complication related to pregnancy or delivery, induced labour, HIV positive, Any other medical complication during pregnancy (excluding chronic hypertension) and incentive for caesarean section (no missing data for this outcome)

**Maternal age, total number of years attended school, birth weight, prelabour rupture of membranes, pregnancy induced hypertension or preeclampsia or eclampsia or chronic hypertension, vaginal bleeding in second half of pregnancy, Referred for complication related to pregnancy or delivery, induced labour, HIV positive, any other medical complication during pregnancy (excluding chronic hypertension), incentive for caesarean section and country (no missing data for this outcome)

†Maternal age, total number of years attended school, primiparaous, birth weight, neonatal death or stillbirth, pregnancy induced hypertension or preeclampsia or eclampsia or chronic hypertension, vaginal bleeding in second half of pregnancy, any antenatal antibiotic treatment, referred for complication related to pregnancy or delivery, induced labour, hiv positive, any other medical complication during pregnancy (excluding chronic hypertension), incentive for caesarean section and country (395 participants with missing data for this outcome)

†† Maternal age, Primiparaous, birth weight, vaginal bleeding in second half of pregnancy, referred for complication related to pregnancy or delivery, any other medical complication during pregnancy (excluding chronic hypertension), country and incentive for caesarean section (501 participants with missing data for this outcome)

^**†††**^Maternal age, total number of years attended school, birth weight, neonatal death or stillbirth, prelabour rupture of membranes, pregnancy induced hypertension or preeclampsia or eclampsia or chronic hypertension, vaginal bleeding in the second half of pregnancy, any antenatal antibiotic treatment, breech or other non-cephalic presentation, referred for complication related to pregnancy or delivery, induced labour, hiv positive, any other medical complication during pregnancy (excluding chronic hypertension), country and incentive for caesarean section (data available for at least one of the included outcomes for all participants)

**Table 4:** Relationship between mode of delivery and severe maternal outcomes by continent

Severe Maternal Outcomes	No (%)	Adjusted OR [95%CI]
**Africa **^**†**^		
Spontaneous (reference)	1011/69,364 (1.46)	1 to
Operative	151/2,298 (6.57)	2.58 (2.12-3.15)
Antepartum without indications	**14/63(22.22)**	71.29 (32.06 to 158.55)
Intrapartum without indications	**36/202(17.82)**	40.67 (24.56-67.33)
Antepartum with indications	**960/2,399(40.02)**	88.61 (74.88 to 104.86)
Intrapartum with indications	**1,942/7,396(26.26)**	54.26 (47.81 to 61.59)
		
**Americas**^**††**^		
Spontaneous (reference)	946/61130 (1.55)	1
Operative	50/1533 (3.26)	2.2 (1.62 to 2.99)
Antepartum without indications	**6/157(3.82)**	1.94 (0.77 to 4.9)
Intrapartum without indications	**10/194(5.15)**	4 (2.05 to 7.82)
Antepartum with indications	**803/13759(5.84)**	3.04 (2.71 to 3.41)
Intrapartum with indications	**640/20120(3.18)**	1.91 (1.71 to 2.13)
		
**Asia**^**†††**^		
Spontaneous (reference)	1190/75057 (1.59)	1
Operative	145/3465 (4.18)	1.91 (1.56 to 2.35)
Antepartum without indications	**8/1515(0.53)**	2.14 (1.04 to 4.43)
Intrapartum without indications	**40/554(7.22)**	12.86 (8.83 to 18.73)
Antepartum with indications	**689/10853(6.35)**	8.09 (7.12 to 9.18)
Intrapartum with indications	**1941/16506(11.76)**	11.61 (10.56 to 12.76)

†Maternal age, Total number of years attended school, Primiparaous, Birth weight, Neonatal death or stillbirth, Caesarean section at last pregnancy, Pregnancy induced hypertension or preeclampsia or eclampsia or chronic hypertension, Suspected fetal growth impairment, Vaginal bleeding in 2nd half of pregnancy, Referred for complication related to pregnancy or delivery, Any other medical complication during pregnancy (excluding chronic hypertension), Country, Incentive for caesarean section

††Birth weight, Prelabour rupture of membranes, Pregnancy induced hypertension or preeclampsia or eclampsia or chronic hypertension, Vaginal bleeding in 2nd half of pregnancy, Any antenatal antibiotic treatment, Referred for complication related to pregnancy or delivery, Induced labour, HIVPOS, Any other medical complication during pregnancy (excluding chronic hypertension) and Incentive for caesarean section

††† Maternal age, Total number of years attended school, Primiparous, Birth weight, Neonatal death or stillbirth, Prelabour rupture of membranes, Pregnancy induced hypertension or preeclampsia or eclampsia or chronic hypertension, Vaginal bleeding in 2nd half of pregnancy, Breech or other non-cephalic precentation, Referred for complication related to pregnancy or delivery, HIVPOS, Any other medical complication during pregnancy (excluding chronic hypertension), Country and Complexity of index

(Differently from the previous Asian specific analysis [7], the severe maternal outcomes included in this analysis are: death, admission to ICU, blood transfusion and hysterectomy)

Table 5 presents the relationship between mode of delivery and severe perinatal outcome. Compared to spontaneous vaginal delivery, operative vaginal delivery, antepartum caesarean section with indications and any intrapartum caesarean section were associated with an increased risk of severe perinatal outcomes. In the subgroup of neonates with breech presentation, caesarean delivery was associated with a reduced risk of severe perinatal outcome (antepartum caesarean section, adj OR = 0.64, 95% CI 0.51 to 0.80; intrapartum caesarean section, adj OR = 0.80, 95% CI 0.66 to 0.97; data not shown in tables).

**Table 5:** Perinatal outcomes among singleton and first child of multiple births by mode of delivery

Perinatal outcomes	n/N (%)	Adjusted OR [95%CI]
**Perinatal mortality**^**†**^		
Spontaneous (reference)	**3218/205551(1.57)**	**1**
Operative	**415/7296(5.69)**	**3.16 (2.76 to 3.61)**
Antepartum without indications	**6/1735(0.35)**	**0.72 (0.26 to 1.97)**
Intrapartum without indications	**16/950(1.68)**	**2.41 (1.39 to 4.2)**
Antepartum with indications	**534/27011(1.98)**	**1.3 (1.15 to 1.47)**
Intrapartum with indications	**1081/44022(2.46)**	**2.01 (1.84 to 2.19)**
		
**Fetal death**^**††**^		
Spontaneous (reference)	**1981/205551 (0.96)**	**1**
Operative	**291/7296 (3.99)**	**3.25 (2.79 to 3.79)**
Antepartum without indications	**3/1735(0.17)**	**0.48 (0.12 to 1.98)**
Intrapartum without indications	**11/950(1.16)**	**2.3 (1.2 to 4.4)**
Antepartum with indications	**248/27011 (0.92)**	**0.92 (0.78 to 1.09)**
Intrapartum with indications	**577/44022 (1.31)**	**1.6 (1.43 to 1.79)**
		
**Early neonatal deaths up to hospital discharge**^**‡**^		
Spontaneous (reference)	**1237/203570 (0.61)**	**1**
Operative	**124/7005 (1.82)**	**2.76 (2.19 to 3.47)**
Antepartum without indications	**3/1732(0.17)**	**0.88 (0.21 to 3.62)**
Intrapartum without indications	**5/939(0.53)**	**2.01 (0.8 to 5.06)**
Antepartum with indications	**286/26477(1.08)**	**1.68 (1.42 to 1.98)**
Intrapartum with indications	**504/43445(1.16)**	**2.2 (1.94 to 2.5)**
		
**Stay for ≥7 days in neonatal intensive care unit‡‡**		
Spontaneous (reference)	**2704/205551(1.32)**	**1**
Operative	**203/7296(2.78)**	**1.57 (1.31 to 1.88)**
Antepartum without indications	**17/1735(0.98)**	**1.18 (0.7 to 1.99)**
Intrapartum without indications	**16/950(1.68)**	**2.09 (1.21 to 3.6)**
Antepartum with indications	**1466/27011(5.43)**	**2.63 (2.4 to 2.89)**
Intrapartum with indications	**1625/44022(3.69)**	**2.77 (2.55 to 3)**
		
**Severe perinatal outcome‡‡‡**		
Spontaneous (reference)	**5820/205551 (2.83)**	**1**
Operative	**613/7296 (8.4)**	**2.33 (2.07 to 2.62)**
Antepartum without indications	**22/1735(1.27)**	**1 (0.61 to 1.62)**
Intrapartum without indications	**32/950(3.37)**	**2.48 (1.66 to 3.69)**
Antepartum with indications	**1968/27011(7.29)**	**2.05 (1.9 to 2.22)**
Intrapartum with indications	**2656/44022(6.03)**	**2.42 (2.27 to 2.58)**

† Maternal age, Total number of years attended school, birth weight, Neonatal death or stillbirth, Pregnancy induced hypertension or preeclampsia or eclampsia or chronic hypertension, Vaginal bleeding in 2nd half of pregnancy, Any antenatal antibiotic treatment, Referred for complication related to pregnancy or delivery, Induced labour, Any other medical complication during pregnancy (excluding chronic hypertension), country and gestational age

†† Maternal age, Total number of years attended school, birth weight, Neonatal death or stillbirth, Pregnancy induced hypertension or preeclampsia or eclampsia or chronic hypertension, Vaginal bleeding in 2nd half of pregnancy, Referred for complication related to pregnancy or delivery, Any other medical complication during pregnancy (excluding chronic hypertension) and gestational age

‡ Total number of years attended school, birth weight, Neonatal death or stillbirth, Vaginal bleeding in 2nd half of pregnancy, Referred for complication related to pregnancy or delivery, Induced labour, country and gestational age

‡‡ Maternal age, Total number of years attended school, Primigravida, birth weight, Neonatal death or stillbirth, Caesarean section at last pregnancy, Prelabour rupture of membranes, Pregnancy induced hypertension or preeclampsia or eclampsia or chronic hypertension, Suspected fetal growth impairment, Any antenatal antibiotic treatment, Breech or other non-cephalic presentation, Referred for complication related to pregnancy or delivery, Induced labour, Any other medical complication during pregnancy (excluding chronic hypertension), country, complexity of index and gestational age

‡‡‡ Maternal age, Total number of years attended school, birth weight, Neonatal death or stillbirth, Caesarean section at last pregnancy, Prelabour rupture of membranes, Pregnancy induced hypertension or preeclampsia or eclampsia or chronic hypertension, Suspected fetal growth impairment, Vaginal bleeding in 2nd half of pregnancy, Breech or other non-cephalic presentation, Referred for complication related to pregnancy or delivery, Induced labour, Any other medical complication during pregnancy (excluding chronic hypertension), country, complexity of index and gestational age

## Discussion

In this paper, the intrinsic risk of caesarean sections was assessed. Caesarean sections were associated with an intrinsic risk for short-term severe maternal outcomes. Overall, this risk was higher in African countries than in Asia or Latin America. These findings corroborate the previous WHO Global Survey continental-level analyses [5-7,15] and also provide further evidence for cautioning physicians and patients about issues related with caesarean sections with no medical indications.

Over the years, it has been noted in the medical literature that the interaction between the maternal underlying condition and the caesarean section operation complicates the assessment of caesarean section intrinsic risk. In order to address this issue, we tried to account for the contribution of the medical conditions leading to the operation, other risk factors, confounders and effect modifiers through multilevel statistical modeling. The results of this statistical adjustment (presented in Table 3) show a substantial positive association between caesarean section and severe maternal outcomes. Similar methods were used in the previous continental analysis for Asia and it is reassuring to find a consistent trend in the two other continents. Furthering this approach, we conducted a subgroup analysis including only women with no identifiable medical risk factors: the caesarean section operation was found to increase the risk of severe maternal outcomes. Stratifying the assessment at continent and country level, similar findings were found. Operative vaginal delivery was also found to be associated with an increased risk of severe maternal outcomes suggesting that these interventions should be performed very carefully by trained providers and only when necessary.

Severe maternal outcomes are relatively rare conditions during pregnancy and childbirth. This factor may contribute to the misperception of safety related to caesarean section and lead to the overuse of the procedure. The relatively low frequency of severe outcomes also makes their appropriate assessment more complex, requiring large databases. The present analysis and findings were possible due to key features of the WHO Global Survey on Maternal and Perinatal Health project. In this project, standard methods were used across 373 hospitals around the world, generating a consistent and large database, containing a considerable number of severe maternal outcomes, including maternal deaths, intensive care unit admission, blood transfusion and hysterectomy. Another unique feature of the database was the concurrent data collection in all participating health facilities in each region thus ensuring that clinical practice in the various settings was captured during the same period. Quality features of the study also ensured that nearly all deliveries in each health facility during the study period were recorded.

Nevertheless, we should acknowledge some weaknesses of this study. The first one is the study design. This is a cross-sectional study, which *per se *makes this evaluation unable to establish causal relationships between caesarean sections and the maternal outcomes. However, considering the existing constraints in terms of using other designs, we consider this a fair approach to the question. Another point is that our findings may not be country or continent representative; our sample was based on a random selection of countries, regions within countries and health facilities. Doing so, we simply aimed at avoiding other selection biases, and tried to be inclusive as much as possible, considering the available resources we had. Another issue that should be considered is the disproportionately higher contribution of Chinese health facilities to the group of women undergoing caesarean sections without medical indications. The "country" variable was included in the statistical model used to adjust the findings, reducing both the clustering effect and the role of an individual country's contribution to the results. On the other hand, in the assessment of caesarean sections with indications, the Chinese facilities' contribution was not especially prominent and the findings were similar.

The low frequency of events makes the absolute risk associated with caesarean sections low, but even this low risk is substantially higher when compared to spontaneous vaginal deliveries. From the population perspective and considering the frequency with which the procedure is practiced, these findings may be relevant for avoiding the occurrence of severe maternal outcomes, especially in those settings where avoidable caesarean sections are more prevalent.

In this context, one could speculate about the relationship of these findings with the underlying health system and the implication of these results to developed countries. The higher intrinsic risk of caesarean sections observed in Africa compared to Asia and Latin America and the lower intrinsic risk in Latin America compared to Asia could suggest some ecological relationship between the strength of health system, urbanization, facility-based care and development status with the safety of surgical procedures including caesarean section. Data from Japan, the only developed country that took part in the study, could clarify that, but the low number of severe maternal outcomes actually prevented a conclusive assessment by country. Nevertheless, the above mentioned ecological relationship could suggest that in developed settings the intrinsic risk of caesarean sections would be lower compared to less developed settings.

## Conclusion

We conclude that caesarean sections should be performed when a clear benefit is anticipated, a benefit that might compensate for the higher costs and additional risks in the context of the specific setting where the operation is taking place. This additional risk should be considered by health care professionals and patients when deciding the mode of delivery. In the end, the main challenge related to caesarean sections is making the best use of this procedure, which is certainly an important resource for the reduction of maternal mortality, but of which overuse may be associated with an increased risk of severe maternal outcomes.

## Abbreviations

95% CI: 95% Confidence Interval; Adj OR: Adjusted Odds Ratio; GLIMMIX: procedure for generalized linear mixed models; GLLAMM: generalized linear and latent mixed models; HRP: UNDP/UNFPA/WHO/World Bank Special Programme of Research, Development and Research Training in Human Reproduction; ICU: Intensive Care Unit; OR: Odds Ratio; UNDP: United Nations Development Programme; UNFPA: United Nations Population Fund; USAID: United States Agency for International Development; WHO: World Health Organization; WHOGS: World Health Organization Global Survey on Maternal and Perinatal Health.

## Competing interests

The authors declare that they have no competing interests.

## Authors' contributions

JPS, AMG, PL and ML conceived this analysis of the WHO Global Survey on Maternal and Newborn Health database. ML carried out the statistical analysis. JPS drafted the manuscript and AMG, PL, ML, GC, BF and PR provided important input on it. All authors read and approved the final manuscript.

## Pre-publication history

The pre-publication history for this paper can be accessed here:

http://www.biomedcentral.com/1741-7015/8/71/prepub
